# Fluctuating Asymmetry and Environmental Stress: Understanding the Role of Trait History

**DOI:** 10.1371/journal.pone.0057966

**Published:** 2013-03-05

**Authors:** Greet De Coster, Stefan Van Dongen, Phillista Malaki, Muchai Muchane, Angelica Alcántara-Exposito, Hans Matheve, Luc Lens

**Affiliations:** 1 Terrestrial Ecology Unit, Ghent University, Ghent, Belgium; 2 Evolutionary Ecology Group, University of Antwerp, Antwerp, Belgium; 3 National Museums of Kenya, Nairobi, Kenya; University of Milan, Italy

## Abstract

While fluctuating asymmetry (FA; small, random deviations from perfect symmetry in bilaterally symmetrical traits) is widely regarded as a proxy for environmental and genetic stress effects, empirical associations between FA and stress are often weak or heterogeneous among traits. A conceptually important source of heterogeneity in relationships with FA is variation in the selection history of the trait(s) under study, i.e. traits that experienced a (recent) history of directional change are predicted to be developmentally less stable, potentially through the loss of canalizing modifiers. Here we applied X-ray photography on museum specimens and live captures to test to what extent the magnitude of FA and FA-stress relationships covary with directional shifts in traits related to the flight apparatus of four East-African rainforest birds that underwent recent shifts in habitat quality and landscape connectivity. Both the magnitude and direction of phenotypic change varied among species, with some traits increasing in size while others decreased or maintained their original size. In three of the four species, traits that underwent larger directional changes were less strongly buffered against random perturbations during their development, and traits that increased in size over time developed more asymmetrically than those that decreased. As we believe that spurious relationships due to biased comparisons of historic (museum specimens) and current (field captures) samples can be ruled out, these results support the largely untested hypothesis that directional shifts may increase the sensitivity of developing traits to random perturbations of environmental or genetic origin.

## Introduction

Fluctuating asymmetry (FA), i.e. small, random deviations from perfect symmetry in otherwise bilaterally symmetrical traits, is widely regarded as an individual-based proxy of environmental and genetic stressors in a variety of taxa (e.g., [1]–[5]). As both sides of bilateral traits develop under control of an identical genome, FA is assumed to reflect the inability of organisms to buffer their development against random perturbations, known as developmental instability (DI), and thereby mirror the level of stress to which they are imposed (reviewed in [6], [7]). Two other types of bilateral asymmetry, directional asymmetry (DA, normal distribution of left minus right trait values with non-zero mean) and antisymmetry (AS, bimodal distribution with zero mean) are believed to have a significant genetic basis [8], [9] and are therefore regarded unsuited as a measure of DI. Despite a strong theoretical framework on FA-stress relationships, the observed associations are often weak, species-, population-, or trait-specific (e.g., [10]–[12]), all of which hamper the use of FA as bioindicator in evolutionary ecology and conservation biology (e.g., [13], [14]). Several conceptual, methodological and statistical issues have been put forward to explain this heterogeneity in FA-stress relationships, including the fact that in many populations the variation in underlying DI may be too low, and the correlation between DI and FA too weak, to reveal differences in DI among individuals (see e.g., [13]–[16] for extensive reviews).

A conceptually important - yet rarely tested - factor that may affect relationships between FA and stress across species and populations is the selection history of the trait under study. The observation that traits under sexual selection (i.e. where strong directional selection is acting within one sex) often show higher levels of FA has triggered the premise that FA in traits under selection may be more sensitive to stress and therefore provide an honest signal of individual quality [17], [18]. The most likely explanation for such increased sensitivity to stress is that the development of traits that experienced a (recent) history of directional selection may become destabilized through the loss of canalizing modifiers ([19]; see [16], [20] for alternative mechanisms). So far, putative effects of selection history on FA-stress associations have mainly been studied within the context of sexual selection. For instance, in bird species with a female preference for the largest male ornament, FA was shown to be inversely related to the absolute size of the ornament, while no (or more complex) relationships were detected in absence of such female preference ([21]). A small number of studies also provided (indirect) evidence for a role of selection history in the strength of relationships between FA, stress and fitness in traits that were not assumed to be under direct sexual selection. For instance, in three-spined stickleback (*Gasterosteus aculeatus*) populations that recently colonized fresh-water habitats, several traits evolved during colonization while others did not show selective directional shifts. Van Dongen et al. [22] showed that FA in these colonizers was inversely related to the amount of genetic variation at neutral markers (a measure of genetic stress) for traits under directional selection, but not for non-evolved ones. In contrast, traits under artificial selection in *Drosophila melanogaster* did not show increased levels of FA, but this experiment was performed under optimal developmental conditions which hampered the study of relationships with environmental stress [20]. A more recent study of *D. bipectinata* populations that had experienced recent directional changes in trait size did reveal significant associations between FA and mating success [16]. While these and other studies explicitly hint towards a role of selection history in the strength of relationships between FA, stress and fitness, there is a clear need for further empirical testing of this hypothesis.

We here study to what extent the magnitude of FA and FA-stress relationships in traits related to the flight apparatus of four tropical rainforest birds from an Eastern Arc Mountains biodiversity hotspot (Taita Hills, SE Kenya) is associated with directional shifts in trait size over the past 60–70 years [23], [24]. The indigenous forest cover in the Taita Hills decreased by ca. 98% over the last 200 years, as a result of agricultural expansion, logging, pole cutting and cattle grazing, and formerly continuous tracts of rainforest became subdivided in small, isolated fragments, most strongly so since the early 1960s [25]–[27]. Based on 18 years of demographic, genetic and dispersal data from eight forest-restricted bird species [28], [29], it was earlier shown that this decrease in landscape connectivity resulted in a significantly loss in mobility over time in some species, while others seemed to cope better, possibly as a result of phenotypic and/or behavioural adaptations ([28]; see also [30], [31]). In addition to landscape-level effects on mobility, species also varied in their sensitivity to patch-level forest degradation, as inferred from historic changes in tarsus FA between museum specimens (collected prior to degradation) and post-degradation live captures from the same localities [32]. Elaborating on these longitudinal data, we here quantify the extent of asymmetric development of wing traits and tarsi in four of these species and test how levels of FA covary with changes in trait size over several decades. Because FA is assumed to increase with growth rate as developmental precision is compromised when more energy is allocated to growth [33], [34], we further test whether traits that increased in size over time develop more asymmetrically compared to those that decreased. Apart from FA, we also modeled relationships with DA, as failure to appropriately account for bilateral asymmetry may skew the distribution of signed asymmetry values and hence violate the assumptions for translating observed patterns of FA into presumed underlying patterns of developmental instability [13].

In addition to standard exterior measurements (tarsus length), we applied X-ray photography to measure FA and DA in bone structures related to the flight apparatus. Bone asymmetry is generally assumed to mirror developmental instabilities more accurately than asymmetry in plumage-related traits (e.g. wing or tail length) that may be subject to substantial wear [35]. As is the case with digital photography [36], X-rays are deemed particularly appropriate when traits can be well-represented in two dimensions. Yet, despite this strong potential, the technique has only been rarely used in studies of natural population (see [37], [38] for examples).

## Materials and Methods

### Ethics Statement

This study was conducted under research permits NCST/5/002/R/274/4 and NCST/RRI/12/1/BS-011/58 of the Kenyan National Council for Science and Technology. Permission to work in the study area was granted by the Taita Taveta District Commissioner, while permission for the export and use of the portable X-ray unit was granted by the Belgian Federal Agency for Nuclear Control. All fieldwork complied with the Belgian and Kenyan ethical guidelines for animal welfare, and all necessary steps were taken to minimize animal suffering during handling. No birds were kept in captivity or injured by any means (no sampling of blood, feathers or other tissues), and all individuals were released in perfect body condition immediately after X-raying. When shipping and handling museum specimens, all regulations specified in the terms of loan were strictly adhered to.

### Study Area and Species

The Taita Hills forest archipelago (SE Kenya, 03°20′S, 38°15′E) currently comprises three indigenous forest fragments (86–220 ha) and eight tiny forest remnants (2–8 ha) that are isolated from other highland forests by ca. 80 km of semi-arid plains [39]. All remnant forests are located at hilltops and ridges, and are separated by small holder cultivation plots and exotic plantation forests [26], [32], [40]. Data for this study were collected in two of the larger fragments, Ngangao (NG, 120 ha) and Chawia (CH, 86 ha) that suffered a 50% and 85% size reduction since the early 1960s, respectively [25]. Based on historic forest cover and vegetation data [32], [41], the smaller fragment has also been more strongly degraded over time. Within both fragments, we trapped and measured individuals of the following four bird species for which adequate numbers of museum specimens were available to allow paired comparisons with live captures: olive sunbird (*Cyanomitra olivacea changamwensis*; OS), Cabanis’s greenbul (*Phyllastrephus cabanisi placidus*; CG), Taita white-eye (*Zosterops (poliogaster) silvanus*; TW) and white-starred robin (*Pogonocichla stellata helleri*; WR).

### Captures, Tarsus Measurements and X-ray Imagery

A total of 210 individuals of the four study species were captured with mist nets in June and July 2009, distributed as follows: fragment CH (OS = 46, CG = 29, TW = 40, WR = 26); fragment NG (OS = 30, CG = 9, TW = 7, WR = 23). Upon capture, tarsus length was measured to the nearest 0.1 mm using digital calipers. To separate bilateral asymmetry from measurement error in mixed regression models (see below), left and right tarsi were measured twice (sequence left-right-left-right or right-left-right-left) with calipers consistently reset to zero after each measurement. To allow unbiased comparisons between live captures and museum specimens (see below), we measured tarsus length from the notch on the back of the intertarsal joint to the lower edge of the last complete scale before the toes diverge [35]. Next, each individual was briefly attached to a rotatable and translatable positioning table with all focal skeletal traits (see below) carefully positioned within a single horizontal plane, and subsequently exposed to 40 kV and 7.2 mAs of continuous current (Mobilux HF 9020 X-ray device with dental DR detector; active area 25.8×36 mm; Verachtert Digital NV) by AA. A small number of individuals were released without being X-rayed to limit capture stress or avoid hypothermia during cold spells, resulting in the following sample size: fragment CH (OS = 23, CG = 13, TW = 26, WR = 13); fragment NG (OS = 22, CG = 9, TW = 5, WR = 18). Next, a total of 51 museum specimens of the four study species (OS = 11; CG = 8; TW = 17; WR = 15) that had been collected by shotgun in fragment NG between 1934–1948 were loaned from the National Museums of Kenya (Nairobi, Kenya), the Museum of Comparative Zoology (Cambridge, USA), the Field Museum of Natural History (Chicago, USA), the Smithsonian Institution (Washington DC, USA), the American Museum of Natural History (New York, USA), and the Natural History Museum (Tring, UK). A small number of specimens had been collected in forest fragment CH too, however, these were not included in our analyses to avoid additional sampling variation. Upon arrival at Ghent University, left and right tarsi of each museum specimen were measured twice following the procedure outlined above. Next, focal skeletal traits were carefully positioned within a single horizontal plane on the rotatable and translatable table of the same portable X-ray device (see higher), and exposed to conditions (40 kV and 7.2 mAs) identical to those used in the field, by the same person (AA).

### Skeletal Measurements

**Figure 1:**
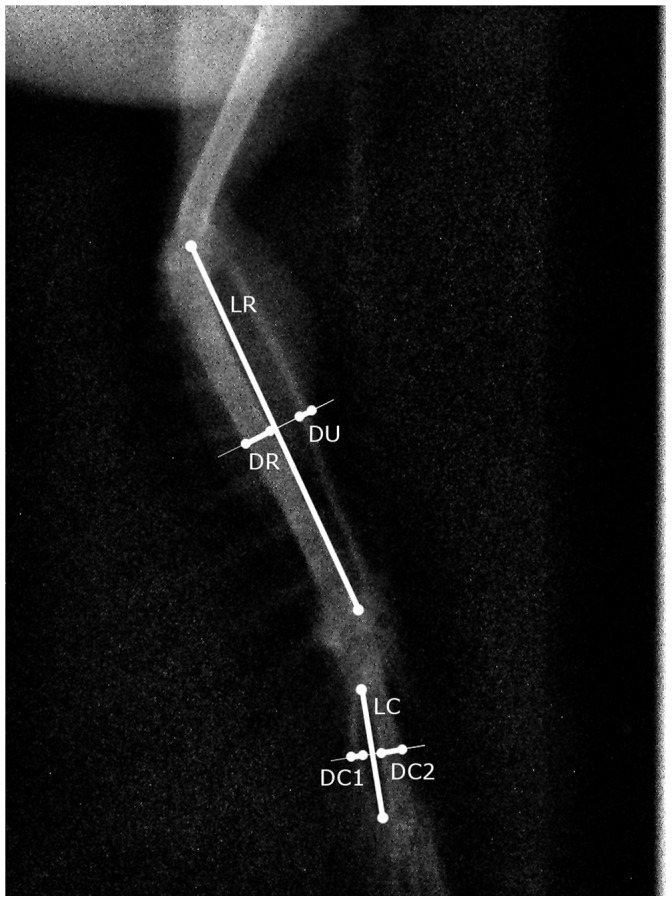
X-ray image of the skeletal structure of a wing with the position of all 12 landmarks and associated wing traits indicated. LR, length radius; DR, diameter radius; DU, diameter ulna; LC, length of the carpometacarpal window; DC1 and DC2, diameter of both bony structures of carpometacarpus.

On each radiograph of the left and right wing of the museums specimens and field captures, a total of 12 landmarks were located with program ImageJ [42] by AA and HM, allowing us to measure six skeletal traits per wing. Selection of these six traits reflected a compromise between optimal representation of the overall wing morphology and maximal repeatability in landmark setting (pilot analysis, data not shown). Landmarks were chosen such that the length of radius-ulna (LR), diameter of radius (DR) and ulna (DU) (through the perpendicular bisector of LR), length of the carpometacarpal window (LC) and diameter of carpometacarpus DC1 and DC2 (through the perpendicular bisector of LC) could be calculated from the coordinates of a minimum number of landmarks (Fig. 1). As landmarks could not be placed unambiguously at the proximal and distal endings of the carpometacarpus, LC was used as a proxy for its total length. X-ray images that did not allow unequivocal location of one or more landmarks were not considered in further analysis, hence final sample sizes were slightly lower than those indicated above. To estimate measurement repeatability, landmarks were placed again on 20 randomly selected individuals, on separate days and blindly with respect to individual and trait side.

### Estimation of FA and Trait Shifts

Individual signed FA values were calculated as the difference between the left and right side of each trait minus its mean population value (calculated as the average left minus right trait values) divided by the mean trait size pooled across sides, per species. By subtracting the mean population value we corrected for DA which may bias FA patterns [8],[9],[43]. We also calculated uncorrected signed asymmetry values to explore relationships with DA directly. To test whether skeletal traits developed independently with respect to bilateral asymmetry, between-trait correlations in signed FA values were explored for all pairwise comparisons. Next, we calculated individual unsigned FA values (i.e. the magnitude of the signed FA; hereafter referred to as “FA”) as the absolute value of each signed FA value. To assess the significance and repeatability of FA, a mixed regression model was fitted to all repeated measurements of left and right trait sides (see [44] for details). Likelihood ratio tests were applied and intraclass correlation coefficients (ICC) were calculated to evaluate the significance and repeatability of FA, respectively. Since FA was positively correlated with trait size (Spearman ρ ≥0.75, p<0.05 for all species), we modeled FA relative to trait size to account for size variation among traits and species [45].

Signed shifts in trait size were calculated for each species*trait combination as the difference between recent (live) minus historic (museum) trait values, relative to the average trait size of the recent samples, in fragment NG. Changes in ‘trait size’ and ‘direction’ were thereby modeled separately (see further). A similar comparison was not possible for populations in fragment CH due to lack of sufficient historic samples (see higher). However, as this fragment has been more severely degraded over time than NG [32], [41], it can be assumed that environmentally-driven changes in trait size were at least as large. To be able to model statistical associations between the magnitude and directionality in trait shifts and trait asymmetry independently, we calculated the absolute value of each signed difference (hereafter referred to as ‘change in trait size’) and modeled a variable ‘direction’ (positive for increased values, negative for decreased values) when explicitly testing for relations with directionality.

While the use of museum collections as a source of baseline data for longitudinal comparisons is well-established, for example through Ellegren et al.'s [46] study of albinistic feathers in *Hirundo rustica* before and after the nuclear accident at Chernobyl, Swaddle et al. [47] urge caution when using museum specimens in studies on FA. Methodological pitfalls include the fact that museum collectors seeking `typical' specimens may be biased towards collecting more symmetrical individuals, and asymmetry due to wear or damage may not be separable from FA. Analyses presented in this study are believed to overcome these problems. First, we only estimated FA in live captures that were not biased towards the largest, most attractive or most symmetrical individuals, whereas museum specimens were exclusively used to estimate directional changes in trait size. Second, specimens from the study area had been collected by shotgun rather than by mist-netting or other techniques that would have allowed selection prior to collection. Moreover, analysis of FA in eight conspicuous feather traits of the same suite of species did not show any correlation with trait size [48]. Third, traits under study both enlarged and decreased over the last 60 years (see Results) which is unlikely to have resulted from unidirectional shrinkage due to the preparation or storage of museum specimens. Finally, throughout the entire study, meticulous care has been taken to use the same portable X-ray device and identical settings, postures and procedures when scanning and measuring museum specimens and live captures (see e.g., [48] for more details on tarsus measurements).

### Statistical Analysis

Statistical associations between the magnitude and directionality in trait shifts and FA were tested with linear mixed models (LMM) with FA as response variable (logarithmic transformed to fulfill the normality assumption of the marginal residuals) and change in trait size, direction, fragment ID and two-factor interactions treated as fixed explanatory variables. As multiple traits measured on a single individual are not statistically independent, individual-specific (i.e. random) intercepts and slopes were included in all LMMs. Degrees of freedom were estimated following Kenward and Roger [49] and a restricted maximum likelihood (REML) procedure was applied in SAS 9.2 (SAS Institute Inc. 2002–2003, Cary, NC, USA) to estimate parameter values. Prior to hypothesis testing, we statistically verified whether measurer identity significantly explained variation in asymmetry or trait shifts. As this was not the case (all p>0.05), measurer ID was not included in subsequent models. Because the number of species with adequate sample sizes was too low to allow robust testing of main or interactive effects with factor species, LMMs were run for each species separately. Whenever appropriate to correct for multiple testing, p-values were adjusted by a standard sequential Bonferroni procedure [50].

## Results

The distribution of bilateral asymmetry values showed no directional component for any of the traits measured (means of signed asymmetry did not differ from zero; Table 1). FA was highly significant for all traits, and levels of within-side measurement error were consistently low compared to between-side differences (repeatability: 0.69< all ICC <0.93; Table 1). Significant between-trait correlation in signed FA was detected in 1 out of 21 cases only, i.e. between the traits DU and LC (Spearman ρ = 0.34, p = 0.001). All other comparisons yielded Spearman values of |ρ| <0.22 (all p>0.14), indicating that our hypothesis testing was not biased by pseudoreplication.

**Table 1:** Descriptive statistics of bilateral asymmetry.

Trait^1^	FA	ME	FA (LR-test)	ICC (%)	mean DA (SE)	DA (t-test)
LR	0.16	0.06	χ^2^ = 11.8, p = 0.0003	73	0.0383 (0.1054)	t_19_ = 0.36, p = 0.72
DR	0.0033	0.0014	χ^2^ = 10.1, p = 0.0007	70	0.0033 (0.0155)	t_19_ = 0.21, p = 0.84
DU	0.0047	0.0013	χ^2^ = 16.5, p<0.0001	78	−0.0115 (0.0174)	t_19_ = −0.66, p = 0.52
LC	0.14	0.017	χ^2^ = 34.1, p<0.0001	89	−0.1435 (0.0891)	t_19_ = −1.61, p = 0.12
DC1	0.0027	0.0012	χ^2^ = 9.7, p = 0.0009	69	0.0058 (0.0140)	t_19_ = 0.41, p = 0.69
DC2	0.0061	0.0014	χ^2^ = 19.9, p<0.0001	81	0.0018 (0.01942)	t_19_ = 0.09, p = 0.93
Tarsus	0.070	0.0055	χ^2^ = 485.8, p<0.0001	93	0.0351 (0.0191)	t_206_ = 1.84, p = 0.068

Levels of fluctuating (FA) and directional (DA) asymmetry relative to measurement error (ME) were obtained from mixed regression model analysis and formed the basis to calculate repeatabilities [intraclass correlation coefficient (ICC) = FA/ (FA+ME)]. While FA was highly significant for all traits (LR-test with distribution being a 50∶50 mixture of and ), none showed significant DA.

1 See Fig.1 for description of trait names.

**Figure 2:**
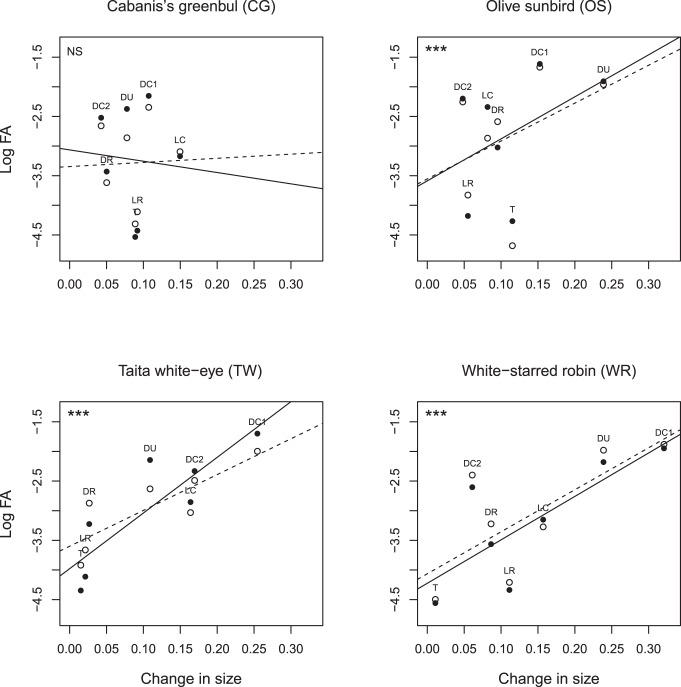
Relationship between change in trait size and log FA of tarsus length and all skeletal traits per species and fragment. Solid dots - solid line: fragment CH; open dots - dashed line: fragment NG. See Fig. 1 for abbreviations of trait names above each dot. Significance levels of both slopes within each subplot are similar (***p<0.001; NS p>0.05).

Signed changes in trait size varied between −32% and +24%, and were significant for all but two traits (p_DC2_ = 0.075, p_tarsus_ = 0.18; all other traits 0.005<p<0.04; one-sample t-tests). The signed magnitude of these changes differed significantly among traits (F_6,21_ = 17.96; p<0.0001; LMM with species as random factor), indicating that some trait values increased in size while others decreased, compared to the historic baseline samples. In all but one species, skeletal traits that underwent larger changes in size over time showed higher levels of FA (OS F_1,309_ = 23.65; p<0.0001; TW F_1,194_ = 117.93; p<0.0001; WR F_1,59.6_ = 60.91; p<0.0001; CG F_1,148_ = 0.0; P = 0.94; Table 2; Fig. 2). The slope of the relationship between change in size on FA did not differ between the two fragments (change in trait size * fragment ID: all p>0.15; Table 2), suggesting that effect sizes were independent of the presumed level of environmental stress.

**Table 2:** Test statistics of fixed explanatory variables in full linear mixed models fitted on log FA measurements of four bird species.

Explanatory variable	CG			OS			TW			WR		
	F	df^1^	p	F	df^1^	p	F	df^1^	p	F	df^1^	P
Change in trait size	0	148	0.94	23.65	309	<0.0001*	117.93	194	<0.0001*	60.91	59.6	<0.0001*
Fragment ID	0.35	148	0.55	0.25	309	0.61	0	28.1	0.99	0.24	158	0.63
Change in trait size* Fragment ID	0.03	147	0.85	0.01	308	0.93	2.13	191	0.15	0.13	56.3	0.72
Change in trait size	0	148	0.95	22.53	309	<0.0001*	127.16	223	<0.0001*	−2	−2	−2
Direction	0	148	0.95	4.15	309	0.04	6.35	223	0.01*	−2	−2	−2
Change in trait size* Direction	0.56	147	0.46	3.68	308	0.06	1.29	222	0.26	18.44	100	<0.0001*

Individual-specific (i.e. random) intercepts and slopes were included in all models, while non-significant interaction terms were removed. Significant p-values are visualized as p<0.05 or **p<0.01,** while * indicates p-values that remained significant after Bonferroni-correction for multiple testing.

1 df refers to degrees of freedom in the denominator (degrees of freedom in the numerator is always 1);

2 not modeled since two-factor interaction was significant.

**Figure 3:**
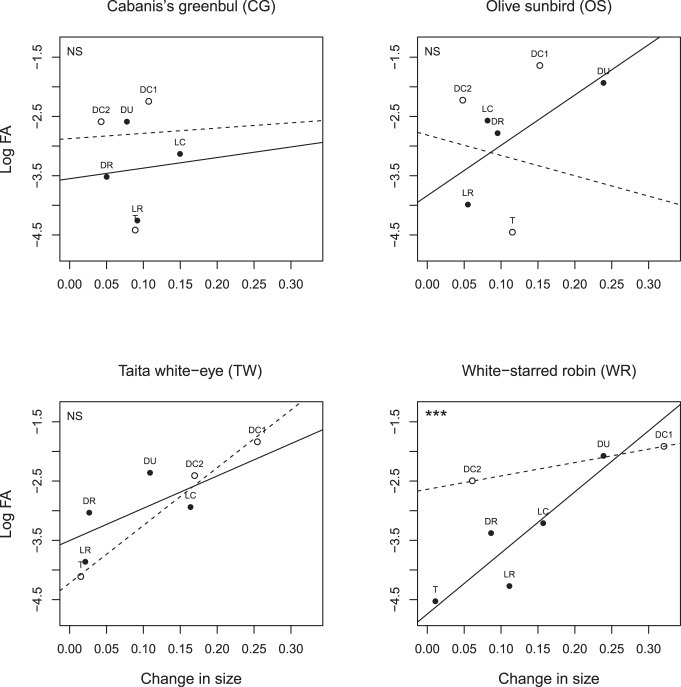
Relationship between change in trait size and log FA of tarsus length and all skeletal traits per species, taking into account the direction of the change.

When correcting for the magnitude of size changes over time, the direction of change was significantly correlated with trait FA in species OS (F_1,309_ = 4.15; p = 0.042) and TW (F_1,223_ = 6.35; p = 0.013; Table 2). In both species, traits that increased in size developed more asymmetrically than traits that decreased in size. In WR, the magnitude and direction of change significant interacted, i.e. levels of trait FA increased more strongly with size change for increasing than for decreasing traits (F_1,100_ = 18.44; p<0.0001; Fig. 3; Table 2), while OS showed a marginally non-significant trend (F_1,308_ = 3.68; p = 0.056; Table 2). In contrast, there was no relation between directionality and FA in species CG (main effect and interaction: both p>0.46; Table 2). Levels of DA were not significantly correlated with changes in trait size for any of the species (all Spearman |ρ| <0.13; all p>0.10), indicating that relationships between FA and changes in size were not confounded by changes in DA.

## Discussion

Here we tested to what extent the rate of precision in wing and leg development in four rainforest birds was related to directional changes in trait size over a sixty years period, by comparing X-ray measurement on museum specimens and recent captures from the same Afrotropical locality. We thereby showed significant variation in both the magnitude and direction of phenotypic change, with some traits increasing in size while others decreased or maintained their original size. In three species, traits that underwent larger directional changes in size were less well buffered against random perturbations during their development, as inferred from their higher levels of fluctuating asymmetry. As predicted, more FA was observed when trait size increased, rather than decreased, over time. As we believe that spurious relationships due to biased comparisons of historic (museum) and current (field) samples can be ruled out (see above), our results support the hypothesis that recent directional changes in trait size increase their sensitivity to random developmental perturbations, regardless of whether these changes result from genetic evolution or reflect phenotypic plasticity [16], [19]. Such association between directional change and stress received hitherto little attention in the literature, in particular for traits that are not considered under direct sexual selection.

Besides the fact that relationships between directional change and trait FA help to understand why more symmetrical individuals are preferred mates and have higher mating success (reviewed in [51]), they may also explain part of the heterogeneity in relationships between FA and stress in conservation studies. To the best of our knowledge, relationships between directional change and FA have never been formally taken into account into the latter. However, indirect evidence for such association stems from a series of studies on a small understory passerine bird of fragmented Brazilian Atlantic rainforest. Rufous gnateaters (*Conopophaga lineata*) that were captured in tiny rainforest fragments showed both larger [30] and more asymmetric [1] wings compared to conspecifics captured in larger forests, and the shift in trait size was interpreted as an adaptation to disperse among poorly-connected forest fragments [30]. While the underlying developmental mechanism(s) remain to be tested in our study (as is the case for most processes related to FA; [6]), the fact that enlarged traits showed higher levels of FA than traits that decreased in size, suggests that high growth rates may have compromised mechanisms controlling early trait development [33], [34].

Yet, larger trait sizes do not necessarily imply higher growth rates as they may also result from increased initial sizes (e.g. by hatching from larger egg; [52]) or from longer time windows during ontogenetic development (reviewed in [33]), and growth rates may also alternate between slow rates and accelerated ‘catch-up’ ones (reviewed in [33]). Such patterns most likely differ between species and traits, and hence also the timing during ontogeny when perturbations can cause aberrant phenotypes. Under these conditions, developmental noise acting randomly on different traits is not expected to cause consistent stress-FA relationships within nor between species, independently of whether the genetic basis of developmental stability itself is trait dependent ([53]; see discussion in [54]). While differences in ontogeny and exposure to developmental perturbations may explain why our four study species varied in relationships with FA, such heterogeneity may also reflect variation in sensitivity to environmental change. In particular, the absence of clear relationships between shifts in trait size and FA in Cabanis’s greenbuls may be explained by the fact that this species only showed minor changes in trait size over time (<15%, see Fig. 2), relative to all other species. Intriguingly, an earlier comparison of past population differentiation (estimated from microsatellite genotypes) with contemporary dispersal rates (estimated from multi-strata capture–recapture models) indicated strong historic mobility loss in this species too [28], possibly reflecting lack of phenotypic plasticity or adaptability to decreased levels of landscape connectivity [26].

Natural populations are globally faced with large-scale habitat alterations that may adversely affect their demographic or genetic population parameters and ultimately cause these populations to go extinct [55]. Identifying such populations of conservation concern before their direct fitness components become irreversibly affected (‘early warning system’ sensu [56]) remains challenging as it requires simple, accurate and cost-effective biomarkers of stress [57]. Over the last decade, FA has recurrently been proposed to operate as an early warning system in a wide variety of taxa (e.g., [1]–[5]). However, results of this study support earlier findings that FA cannot simply be applied as a general predictor of environmental or genetic stress without carefully considering evolutionary, ecological and methodological assumptions (e.g., [10], [11]). Hence, similar to recent recommendations that conservation practices based solely upon current population abundances or movements may, in the long term, prove to be inadequate [28], the use of FA as a bio-indicator in conservation biology may be equally inappropriate if trait histories are not properly taken into account.
